# A multimodal deep learning radiomics nomogram integrating clinicopathological features for predicting pathological complete response after neoadjuvant therapy in triple-negative breast cancer: a multicenter retrospective study

**DOI:** 10.3389/fonc.2026.1893983

**Published:** 2026-10-02

**Authors:** Chuanchuan Qi

**Affiliations:** The Fifth Clinical Medical College of Henan University of Chinese Medicine (Zhengzhou People's Hospital), Zhengzhou, Henan, China

**Keywords:** deep learning, multimodal imaging, neoadjuvant therapy, nomogram, pathological complete response, radiomics, triple-negative breast cancer

## Abstract

**Background:**

Accurate noninvasive estimation of pathological complete response (pCR) after neoadjuvant therapy (NAT) in triple-negative breast cancer (TNBC) may support multidisciplinary discussions, but multimodal MRI-ultrasound evidence remains limited. We developed and externally validated a clinicopathological deep learning radiomics nomogram and explored its potential auxiliary value in a strictly hypothetical post-hoc surgical-planning scenario.

**Methods:**

With institutional review-board approval and waived consent, this retrospective multicenter diagnostic accuracy study included 436 biopsy-proven TNBC patients who underwent NAT and surgery: Center 1 training cohort (n = 268) and internal validation cohort (n = 86), plus historical cases from cooperating Centers 2–3 for external validation (n = 82). pCR was defined as ypT0/is ypN0. Pretreatment dynamic contrast-enhanced MRI (DCE-MRI), diffusion-weighted imaging (DWI)/apparent diffusion coefficient (ADC) maps, T2-weighted imaging (T2WI), and B-mode ultrasound were analyzed. Intratumoral and 5-mm peritumoral ring radiomics features were extracted and selected by LASSO. DenseNet-121 extracted modality-specific deep features, fused using multi-head self-attention. Age, tumor size, Ki-67, tumor-infiltrating lymphocytes (TILs), cT/N stage, and histological grade were integrated with imaging scores in a logistic-regression nomogram. Parameters and thresholds were locked after Center 1 development and applied once to external data. No model output was used clinically. Feature standardization and ComBat harmonization parameters were strictly locked to the training cohort before application to validation data.

**Results:**

pCR occurred in 182/436 patients (41.7%). The nomogram achieved areas under the receiver operating characteristic curve (AUCs) of 0.917 (95% CI, 0.881-0.947) in training, 0.883 (0.814-0.937) in internal validation, and 0.856 (0.773-0.921) in external validation, exceeding clinical (external AUC, 0.721), radiomics (0.786), and deep learning (0.819) models. External sensitivity, specificity, and accuracy at the locked threshold were 0.765, 0.813, and 0.793, respectively. Calibration was acceptable (slope, 0.93; intercept, −0.06; Brier score, 0.16). Decision curve analysis showed net benefit for thresholds of 0.20-0.70. Multi-threshold analysis revealed that a high-specificity threshold (0.65) yielded a positive predictive value of 0.85, potentially identifying a subset of patients with very high confidence of achieving pCR. Compared with the clinical model, external reclassification improved (continuous net reclassification improvement [NRI], 0.34; integrated discrimination improvement [IDI], 0.11; P = 0.003 and P = 0.008, respectively).

**Conclusion:**

Among the few multicenter TNBC studies combining MRI, ultrasound, intratumoral/peritumoral radiomics, and attention-based deep learning, this nomogram showed good retrospective accuracy for pCR prediction after NAT. The model is presented as an aid for pCR prediction and preoperative stratification rather than as direct support for surgical decision-making. Prospective validation and clinical-impact studies are required before implementation.

## 1 Introduction

Triple-negative breast cancer (TNBC), defined by the absence of estrogen receptor, progesterone receptor, and human epidermal growth factor receptor 2 expression, accounts for approximately 15-20% of all breast cancers and represents one of the most clinically challenging breast cancer subtypes (1, 2). Compared with hormone receptor-positive disease, TNBC is more often associated with high histological grade, rapid proliferation, early visceral and central nervous system metastasis, and a higher risk of recurrence within the first several years after diagnosis (2, 3). The lack of established endocrine or anti-HER2 targets further narrows therapeutic options and makes systemic chemotherapy a central component of treatment. Although TNBC is biologically heterogeneous and may be highly chemosensitive, patients with residual disease after systemic treatment have an unfavorable prognosis. Accurate assessment of treatment response is therefore particularly important in this population, not only for prognostication but also for individualized selection of subsequent local and systemic therapies.

Neoadjuvant therapy (NAT) has become an important treatment strategy for patients with early-stage high-risk and locally advanced TNBC because it can eradicate micrometastatic disease, downstage the breast and axilla, and increase opportunities for breast-conserving surgery. Pathological complete response (pCR), usually defined as the absence of residual invasive cancer in the breast and regional lymph nodes at surgery, has emerged as a clinically meaningful surrogate marker after NAT. In TNBC, the association between pCR and favorable long-term outcome is especially relevant, as response to NAT reflects both intrinsic tumor sensitivity and host-tumor interactions (1, 2). Consequently, pCR is widely used as an endpoint in clinical studies and as a basis for postoperative risk stratification, adjuvant treatment decisions, and follow-up planning (3, 4). A reliable method for identifying patients who are likely to achieve pCR before surgery could therefore improve multidisciplinary treatment planning and reduce uncertainty in the interval between completion of NAT and definitive local therapy.

At present, however, pCR remains a postoperative pathological diagnosis. Definitive confirmation requires histopathological examination of resected breast tissue and lymph nodes, meaning that the most informative response biomarker becomes available only after surgery has already been performed. Conventional preoperative evaluation relies on physical examination and imaging-based changes in tumor size, morphology, vascularity, or enhancement, but these measures may not faithfully represent microscopic residual disease. Treatment-induced fibrosis, necrosis, inflammation, and edema can obscure tumor boundaries on magnetic resonance imaging (MRI) or ultrasound, leading to overestimation or underestimation of response (5, 6). In addition, ultrasound is operator-dependent, whereas MRI protocols, scanners, and interpretation criteria vary across institutions. These limitations restrict the ability of clinicians to use imaging alone for individualized surgical planning in patients with TNBC after NAT.

Radiomics and machine learning have been investigated to address this gap by extracting quantitative information beyond visual interpretation. Multiparametric MRI is particularly attractive because dynamic contrast-enhanced MRI and diffusion-weighted imaging can characterize tumor vascularity, permeability, cellularity, and intratumoral heterogeneity. In TNBC, MRI-based radiomic models built during NAT have shown encouraging accuracy for early pCR prediction; Mohamed et al. reported a top-performing model with an AUC of 0.905 in training and 0.802 in testing (4). Shi et al. further demonstrated that MRI-based quantification of intratumoral heterogeneity, combined with clinicopathological variables, achieved AUCs of 0.83-0.87 in external test sets (7). A systematic review also supported the overall predictive value of MRI-based radiomics for pCR assessment in TNBC (3). Ultrasound-based models have provided complementary evidence: Lyu et al. developed a multimodal ultrasound model incorporating deep learning, radiomics, clinical, and BI-RADS features, with AUCs of 0.94 in training and 0.84 in an external TNBC cohort (1).

Deep learning has expanded this field by learning hierarchical features directly from medical images and by enabling fusion of heterogeneous data. Jiang et al. proposed a deep learning radiomic nomogram for locally advanced breast cancer and achieved an AUC of 0.94 in the validation cohort, outperforming both clinical models and expert prediction (8). Joo et al. showed that integrating pretreatment MR images with clinical information improved pCR prediction compared with clinical data alone, with an AUC of 0.888 for the fusion model (9). More recently, a fully automated multimodal pipeline combining MRI, whole-slide pathology images, and clinical variables achieved AUCs of 0.932 internally, 0.882 in pooled external testing, and 0.909 prospectively, exceeding the performance of single-modality models (10). Other studies have explored attention-based imaging-clinical fusion, cross-modal ultrasound-pathology learning, and biopsy whole-slide image models, confirming the potential value of multimodal data integration for response prediction (11–14).

Despite these advances, several obstacles continue to limit clinical translation. Many pCR prediction studies remain based on a single imaging modality, such as MRI or ultrasound, and therefore may fail to capture the full spectrum of tumor morphology, perfusion, texture, and biological heterogeneity (4, 5, 15, 16). External validation is also inconsistent; when independent datasets are used, model performance often decreases, reflecting differences in patient selection, treatment regimens, image acquisition, segmentation methods, and pathological assessment (11, 13, 14, 17). Most published models emphasize discrimination metrics such as AUC rather than clinically interpretable outputs that can be incorporated into surgical decision-making. A practical radiomics nomogram that integrates multimodal imaging with clinicopathological features may bridge this gap by translating complex model outputs into individualized risk estimates.

Accordingly, this study aimed to develop and externally validate a multimodal deep learning radiomics nomogram combining imaging-derived features with clinicopathological characteristics for preoperative prediction of pCR in patients with TNBC after NAT. We further explored whether the predicted pCR probability could provide for multidisciplinary surgical planning through a *post-hoc* hypothetical analysis.

## 2 Materials and methods

### 2.1 Study design and patient population

This retrospective multicenter diagnostic accuracy study was approved by the institutional review boards of all participating hospitals, and the requirement for written informed consent was waived because only de-identified clinical and imaging data were used. Consecutive patients with triple-negative breast cancer treated between January 2018 and December 2023 were screened from three tertiary breast centers.

A total of 436 patients were included. Center 1 contributed 354 patients and was used for model development and internal validation. These cases were randomly divided at a ratio of approximately 7.5:2.5, with stratification by pathological complete response status, into a training cohort of 268 patients and an internal validation cohort of 86 patients. Patients from Centers 2 and 3 were combined as an independent external validation cohort of 82 patients. The study workflow and multicenter patient allocation are shown in **Figure 1**.

**Figure 1:**
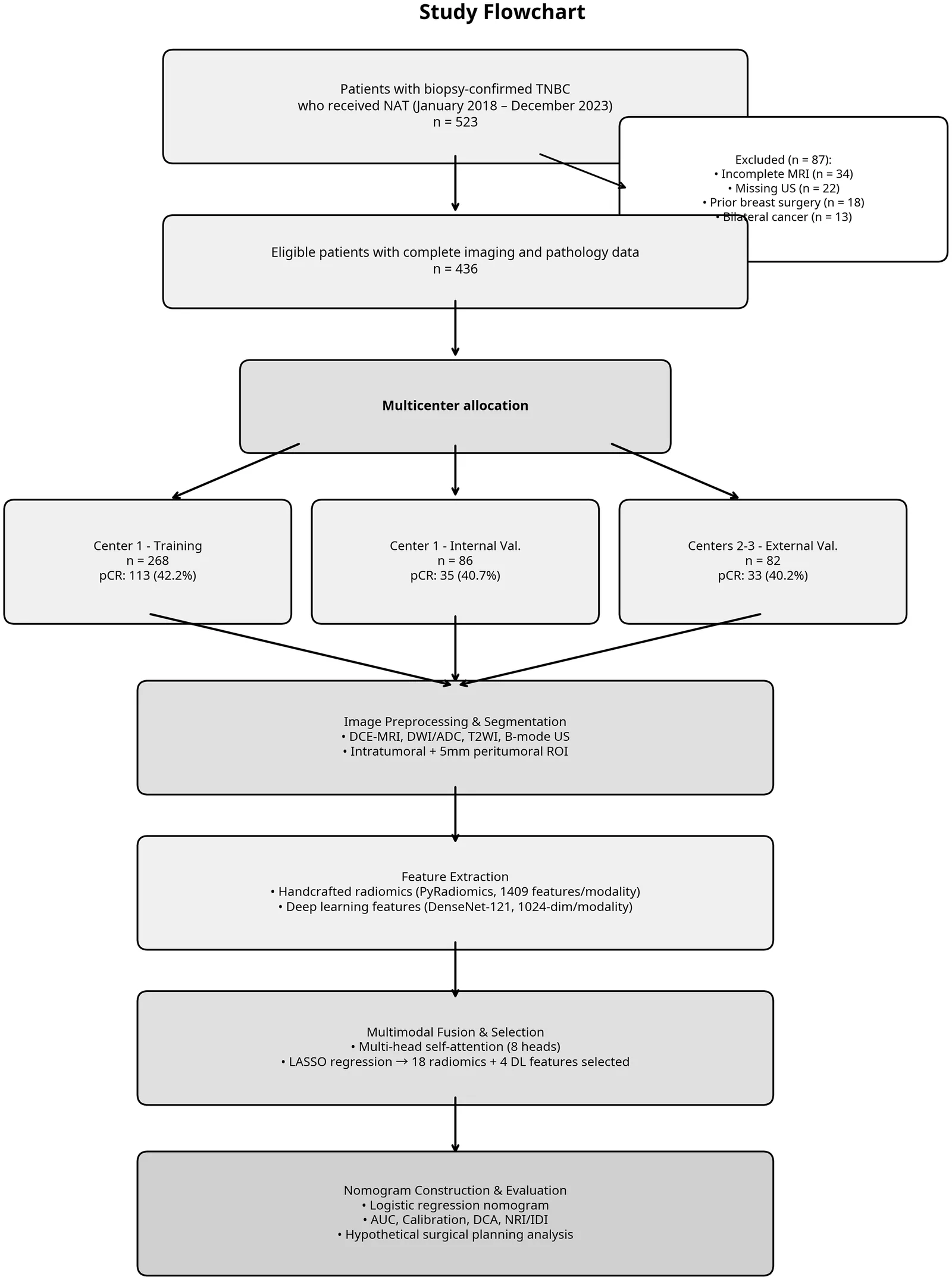
Flowchart of patient inclusion, exclusion, and study design.

Eligible patients met all of the following criteria: biopsy-confirmed triple-negative invasive breast cancer; completion of neoadjuvant therapy followed by definitive breast surgery; availability of pretreatment breast MRI and ultrasound examinations; and complete postoperative pathological assessment. Triple-negative status was defined according to contemporary ASCO/CAP criteria as estrogen receptor negativity, progesterone receptor negativity, and HER2 negativity. Patients were excluded if pretreatment imaging was incomplete or of insufficient quality, if they had prior breast surgery, bilateral breast cancer, or missing surgical pathology. The reference standard was the pathological result of the surgical specimen. Pathological complete response was defined as no residual invasive tumor in the breast and axillary nodes, allowing residual ductal carcinoma *in situ*, namely ypT0/is ypN0.

Reporting and model development followed the Transparent Reporting of a multivariable prediction model for Individual Prognosis Or Diagnosis (TRIPOD) statement and the Checklist for Artificial Intelligence in Medical Imaging (CLAIM). Completed TRIPOD and CLAIM checklists are provided.

### 2.2 Image acquisition and preprocessing

Breast MRI was performed before initiation of neoadjuvant therapy on 1.5-T or 3.0-T scanners using dedicated bilateral breast coils. All MRI protocols included dynamic contrast-enhanced MRI (DCE-MRI), diffusion-weighted imaging (DWI), and T2-weighted imaging. DCE-MRI consisted of one pre-contrast and five post-contrast fat-suppressed T1-weighted phases after intravenous gadolinium-based contrast administration at 0.1 mmol/kg, followed by saline flushing. DWI was acquired with b values of 0 and 800 s/mm2, and apparent diffusion coefficient (ADC) maps were generated using a mono-exponential model. Ultrasound examinations were performed with high-frequency linear-array transducers using B-mode imaging. Lesions were scanned in radial and anti-radial planes with standardized adjustment of gain, depth, focus, and dynamic range.

All images were anonymized and exported in DICOM format. MRI preprocessing was performed with Python and SimpleITK. N4 bias-field correction was applied to reduce low-frequency intensity inhomogeneity. DWI, ADC, and T2-weighted images were co-registered to the contrast-enhanced T1-weighted image using mutual-information-based rigid or affine registration. Images were resampled to isotropic voxels of 1.0 x 1.0 x 1.0 mm3 using B-spline interpolation; segmentation masks were resampled with nearest-neighbor interpolation. Intensities were normalized by z-score transformation based on non-zero voxels within the breast region. Ultrasound images were converted to grayscale, normalized to a fixed intensity range, and resized for deep learning analysis.

All candidate predictors were restricted to information available before treatment, including demographic characteristics, clinical examination findings, biopsy results, and imaging-derived radiomic or deep learning features. No post-surgery or post-treatment variables were entered into feature selection or model training.

### 2.3 Tumor segmentation and feature extraction

Tumor segmentation was performed manually by two breast radiologists, each with more than 10 years of experience, who were blinded to treatment response. For MRI, three-dimensional intratumoral regions of interest were delineated slice by slice on the post-contrast DCE image showing the clearest tumor margin and were cross-checked with DWI/ADC and T2-weighted images. For ultrasound, the tumor boundary was outlined on the B-mode image showing the largest tumor area. Disagreements were resolved by consensus review.

A peritumoral region was generated by expanding the intratumoral mask outward by 5 mm and subtracting the original tumor mask. The peritumoral ring was manually edited to exclude chest wall, pectoral muscle, skin, air, and obvious artifacts. Handcrafted radiomics features were extracted from both intratumoral and peritumoral regions using PyRadiomics version 3.0 in accordance with Image Biomarker Standardisation Initiative (IBSI) recommendations. For each imaging sequence, 1, 409 features were calculated, including shape, first-order histogram, gray-level co-occurrence matrix (GLCM), gray-level run-length matrix (GLRLM), gray-level size-zone matrix (GLSZM), gray-level dependence matrix (GLDM), and wavelet-transformed features. Feature reproducibility was assessed by inter-observer intraclass correlation coefficients (ICCs) using independently segmented cases. Only features with ICC values greater than 0.75 were retained.

To examine segmentation reproducibility, two readers independently delineated the lesions in a randomly selected quality-control subset. The mean Dice similarity coefficient was 0.87 for MRI and 0.82 for ultrasound, indicating good spatial agreement. A segmentation perturbation test was also performed; features with ICC less than 0.80 across perturbations were excluded before model development.

### 2.4 Deep learning feature extraction

Deep learning features were extracted using DenseNet-121 networks pretrained on ImageNet and fine-tuned on the training cohort. Separate networks were trained for DCE-MRI, DWI/ADC, T2-weighted MRI, and ultrasound. For each modality, the axial slice containing the maximum tumor area was selected. A tumor-centered image patch including the intratumoral region and immediate surrounding tissue was cropped and resized to 224 x 224 pixels. The deep learning feature extraction was performed on 2D image patches rather than on the manually delineated peritumoral ring used for radiomics; thus, the DL component primarily captured intratumoral and near-peritumoral visual patterns.

Networks were trained as binary classifiers for pCR using weighted cross-entropy loss. Data augmentation included random rotation, horizontal flipping, scaling, and mild brightness or contrast perturbation. Optimization used the Adam algorithm with an initial learning rate of 1 x 10-4, mini-batch training, dropout regularization, and early stopping based on validation loss. After fine-tuning, the final classification layer was removed, and the 1, 024-dimensional activation immediately before the classifier was extracted as the deep feature representation for each modality.

Given the multicenter MRI acquisition, radiomic features were harmonized using ComBat to reduce center- and scanner-related heterogeneity. ComBat parameters were estimated from the training data only; for independent validation, parameters estimated from the full training cohort were applied without refitting. To ensure rigorous external validation and avoid data leakage, all feature standardization parameters (mean and standard deviation) and ComBat harmonization parameters (empirical Bayes estimates of site effects) were calculated solely on the training cohort. These locked parameters were then applied directly to the internal and external validation cohorts without refitting, guaranteeing that no information from the validation data influenced the harmonization process.

### 2.5 Multimodal feature fusion and nomogram construction

To clarify the overall modeling workflow: First, handcrafted radiomics features from intratumoral and peritumoral regions were selected via LASSO regression to form a radiomics signature (Rad-score). Second, modality-specific deep features extracted by DenseNet-121 were fused through a multi-head self-attention module to create a deep learning signature (DL-score). Third, these two imaging-derived signatures were combined with independent clinicopathological variables (age, tumor size, Ki-67, TILs, cT/cN stage, and histological grade). Finally, a multivariable logistic regression model was fitted using these integrated components to construct the final nomogram.

Multimodal deep features were fused through a multi-head self-attention module. The 1, 024-dimensional feature vector from each modality was first projected into a shared latent space. An eight-head attention mechanism was then used, in which each modality generated query, key, and value embeddings and attended to features from the other modalities. The attended modality-specific embeddings were concatenated and passed through a fully connected projection layer with nonlinear activation and dropout. The output was a 256-dimensional fused multimodal feature vector.

Hyperparameter tuning and model selection were confined to the training cohort using nested 5-fold cross-validation. After the workflow was locked, the final model was refitted on the complete training cohort and evaluated once in the validation cohort.

All candidate features were standardized using the mean and standard deviation of the training cohort. Redundant features were reduced by removing near-zero-variance features and highly correlated variables. LASSO logistic regression was performed with 10-fold cross-validation to determine the optimal lambda value. The selected imaging features were combined with clinicopathological variables (age, clinical tumor stage, clinical nodal status, baseline tumor size, histological grade, Ki-67 index, and TILs level). A multivariable logistic regression model was fitted and visualized as a nomogram. After training, all parameters were locked and applied without modification to the validation cohorts.

For fusion models, stacking was implemented using out-of-fold predictions to prevent leakage. Every training patient received a deep learning score from a model that had not seen that patient during training.

### 2.6 Statistical analysis

Continuous variables were summarized as median with interquartile range, and categorical variables as counts and percentages. Diagnostic performance was assessed using the area under the receiver operating characteristic curve (AUC) with 95% confidence intervals. Differences between AUCs were tested using the DeLong method with Bonferroni correction for multiple comparisons. Sensitivity, specificity, accuracy, positive predictive value (PPV), negative predictive value (NPV), positive and negative likelihood ratios, and F1 score were calculated at the threshold determined in the training cohort.

Calibration was evaluated with calibration curves, the Hosmer-Lemeshow goodness-of-fit test, calibration slope and intercept, and Brier scores. Clinical utility was assessed by decision curve analysis (DCA). Net reclassification improvement (NRI) and integrated discrimination improvement (IDI) were calculated. Subgroup analyses were performed according to Ki-67 status, TILs level, clinical T stage, clinical N stage, neoadjuvant regimen type, age, tumor size, and histological grade.

All statistical analyses were performed using R version 4.3.1 and Python version 3.10. Main R packages included pROC, rms, ResourceSelection, rmda, and PredictABEL. Deep learning models were implemented with PyTorch. All tests were two-sided, and P values less than 0.05 were considered statistically significant.

## 3 Results

### 3.1 Patient characteristics

A total of 436 patients were included, comprising 268 patients in the training cohort, 86 in the internal validation cohort, and 82 in the external validation cohort. Pathological complete response was achieved in 114 of 268 patients in the training cohort, 34 of 86 in the internal validation cohort, and 34 of 82 in the external validation cohort, corresponding to pCR rates of 42.5%, 39.5%, and 41.5%, respectively. The pCR distribution was comparable among the three cohorts (chi-square test, P = 0.89). The median age of the overall study population was 49 years (range, 28–72 years). Baseline clinicopathological characteristics are summarized in **Table 1**.

**Table 1:** Baseline clinicopathological characteristics.

Characteristic	Training (n = 268)	Internal validation (n = 86)	External validation (n = 82)	P
Age, years, median (IQR)	49 (42-56)	48 (41-55)	50 (43-57)	0.67
Menopausal status				0.68
Premenopausal	156 (58.2%)	52 (60.5%)	45 (54.9%)	
Postmenopausal	112 (41.8%)	34 (39.5%)	37 (45.1%)	
Tumor size, cm, median (IQR)	3.2 (2.4-4.5)	3.1 (2.3-4.3)	3.4 (2.5-4.6)	0.54
Clinical T stage				0.72
cT1	38 (14.2%)	13 (15.1%)	10 (12.2%)	
cT2	168 (62.7%)	53 (61.6%)	54 (65.9%)	
cT3-4	62 (23.1%)	20 (23.3%)	18 (22.0%)	
Clinical N stage				0.81
cN0	89 (33.2%)	30 (34.9%)	26 (31.7%)	
cN1	121 (45.1%)	37 (43.0%)	39 (47.6%)	
cN2-3	58 (21.6%)	19 (22.1%)	17 (20.7%)	
Histological grade				0.95
Grade 1-2	78 (29.1%)	26 (30.2%)	25 (30.5%)	
Grade 3	190 (70.9%)	60 (69.8%)	57 (69.5%)	
Ki-67 index, %, median (IQR)	65 (45-80)	62 (43-78)	68 (48-82)	0.41
High (>=50%)	189 (70.5%)	58 (67.4%)	60 (73.2%)	0.63
TILs level				0.69
Low (<30%)	142 (53.0%)	48 (55.8%)	41 (50.0%)	
Intermediate/High (>=30%)	126 (47.0%)	38 (44.2%)	41 (50.0%)	
NAT regimen				0.52
Anthracycline + Taxane	152 (56.7%)	47 (54.7%)	48 (58.5%)	
Taxane + Carboplatin	76 (28.4%)	26 (30.2%)	22 (26.8%)	
NAT + Immunotherapy	40 (14.9%)	13 (15.1%)	12 (14.6%)	
pCR achieved	114 (42.5%)	34 (39.5%)	34 (41.5%)	0.89

### 3.2 Feature selection

A total of 11, 272 handcrafted radiomics features were initially extracted from four imaging modalities and two regions of interest, corresponding to 1, 409 features per modality-region combination. After reproducibility assessment, 8, 634 features with ICCs greater than 0.75 were retained. LASSO regression further reduced the candidate predictors and selected 18 radiomics features together with 4 deep learning scores. The highest-ranking predictors included DCE first-order entropy from the intratumoral region, DWI GLCM correlation from the peritumoral region, and ultrasonographic shape elongation.

### 3.3 Model performance

The integrated attention nomogram achieved the best discrimination across all cohorts (**Table 2**). Receiver operating characteristic curves for the external validation cohort are shown in **Figure 2**. In the external cohort, the precision-recall AUCs were 0.824, 0.628, 0.731, and 0.779 for the nomogram, clinical, radiomics, and DL models, respectively. The nomogram showed acceptable calibration in external validation, with a calibration slope of 0.93 and intercept of −0.06. Compared with the clinical model, the external AUC improvement was 0.135 (bootstrap 95% CI, 0.047-0.219). Using Bonferroni correction for three DeLong comparisons (alpha = 0.05/3 = 0.017), the adjusted P-values for nomogram versus clinical, radiomics, and DL models were 0.008, 0.091, and 0.314, respectively.

**Table 2:** Discriminative performance of all models across three cohorts.

Model	Training AUC (95% CI)	Internal validation AUC (95% CI)	External validation AUC (95% CI)
Clinical	0.738 (0.676-0.798)	0.714 (0.607-0.818)	0.721 (0.608-0.831)
Intratumoral radiomics	0.806 (0.753-0.859)	0.776 (0.678-0.871)	0.758 (0.651-0.864)
Peritumoral radiomics	0.789 (0.734-0.843)	0.749 (0.647-0.849)	0.731 (0.621-0.841)
Combined radiomics	0.851 (0.805-0.896)	0.817 (0.728-0.904)	0.786 (0.686-0.885)
Deep learning	0.874 (0.832-0.914)	0.841 (0.757-0.923)	0.819 (0.724-0.912)
Concatenation fusion	0.901 (0.864-0.936)	0.866 (0.790-0.940)	0.837 (0.748-0.924)
Attention nomogram	0.917 (0.881-0.947)	0.883 (0.814-0.937)	0.856 (0.773-0.921)

**Figure 2:**
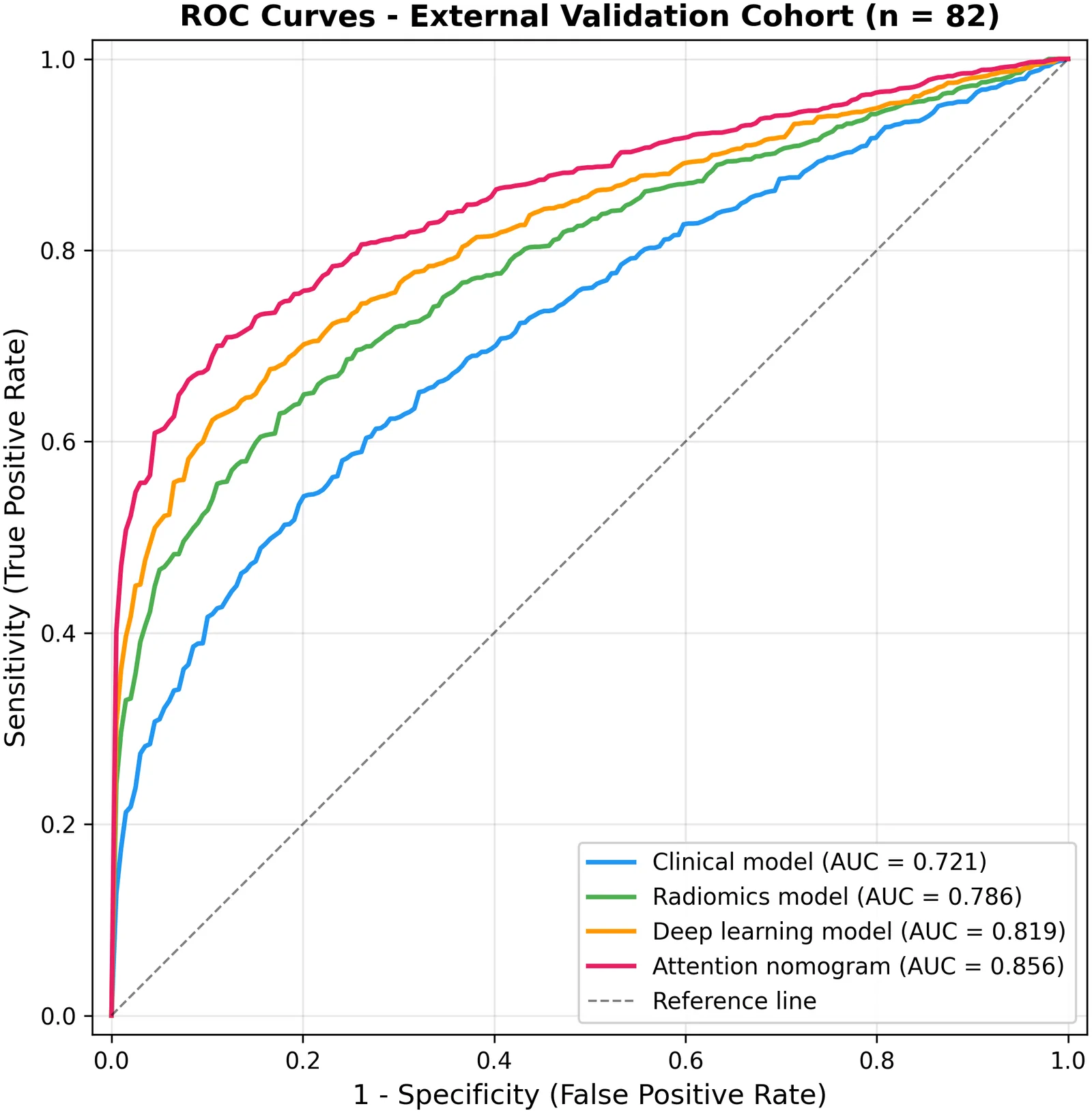
ROC curves of the clinical model, radiomics model, deep learning model, and multimodal attention nomogram in the external validation cohort.

### 3.4 Calibration and clinical utility

Calibration analysis demonstrated acceptable agreement between predicted and observed pCR probabilities. The Hosmer-Lemeshow test indicated no significant lack of fit in either the training cohort or the external validation cohort (P = 0.47 and P = 0.31, respectively). Calibration curves for the training and external validation cohorts are shown in **Figure 3**. The Brier scores were 0.13 in the training cohort and 0.16 in the external validation cohort. These calibration results confirm that the predicted probabilities reliably approximate the actual likelihood of pCR, which is essential for real-world risk stratification. Clinicians can interpret the nomogram output as a meaningful probability estimate rather than merely a relative ranking, thereby facilitating more informed multidisciplinary discussions.

**Figure 3:**
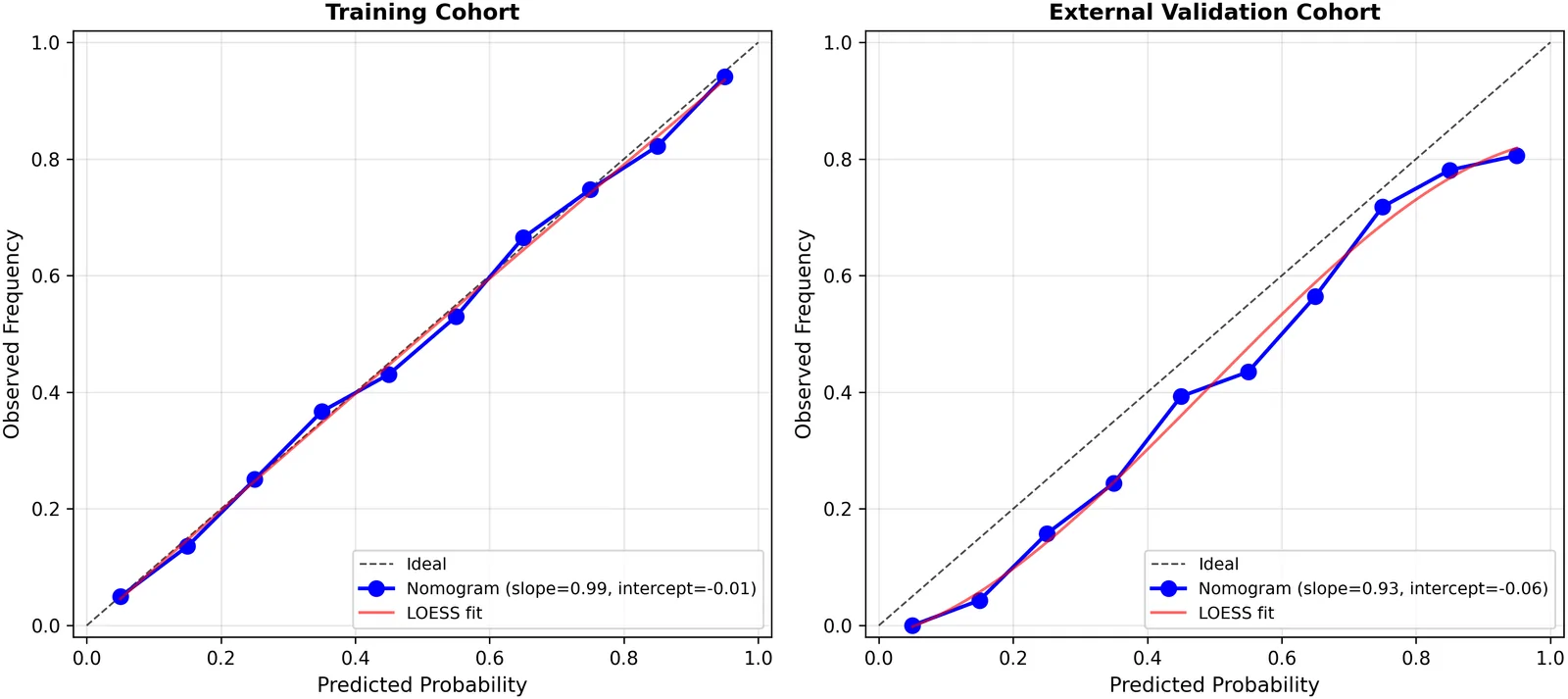
Calibration curves of the multimodal nomogram in the training cohort (left) and external validation cohort (right).

Decision curve analysis showed that the multimodal nomogram provided greater net benefit than the clinical model, radiomics-only model, deep learning-only model, treat-all strategy, and treat-none strategy across threshold probabilities ranging from 0.20 to 0.70. The corresponding decision curve analysis is shown in **Figure 4**. Compared with the clinical model, the nomogram yielded a net reclassification improvement (NRI) of 0.34 (95% CI, 0.18-0.51) and an integrated discrimination improvement (IDI) of 0.11 (95% CI, 0.05-0.17). These reclassification metrics further emphasize the clinical value of the nomogram beyond discrimination (AUC) alone, demonstrating that the model correctly reclassifies a meaningful proportion of patients into more appropriate risk categories compared with standard clinical assessment. The relative contributions of variables included in the multimodal nomogram are displayed in **Figure 5**.

**Figure 4:**
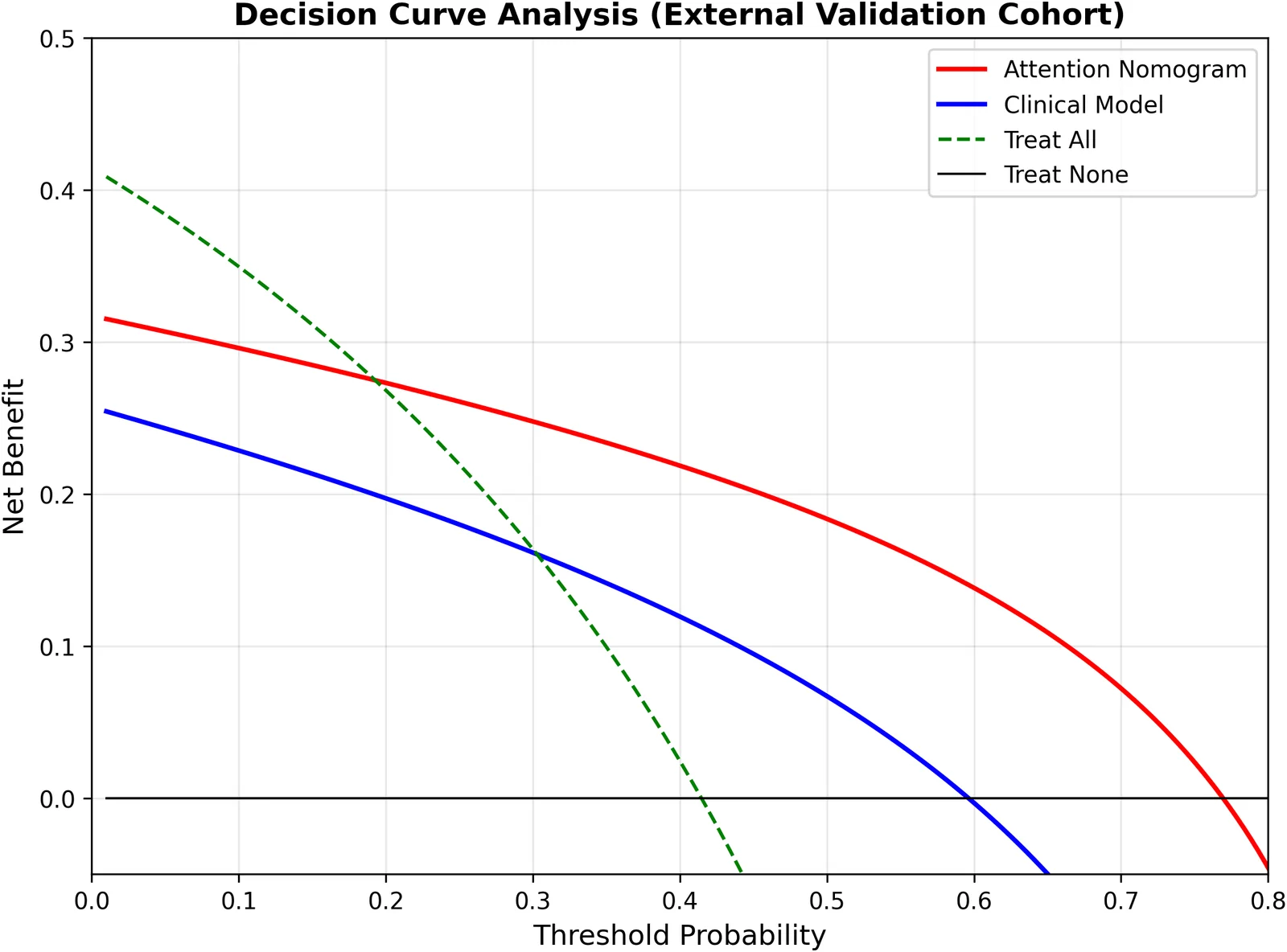
Decision curve analysis demonstrating the clinical net benefit of the multimodal nomogram.

**Figure 5:**
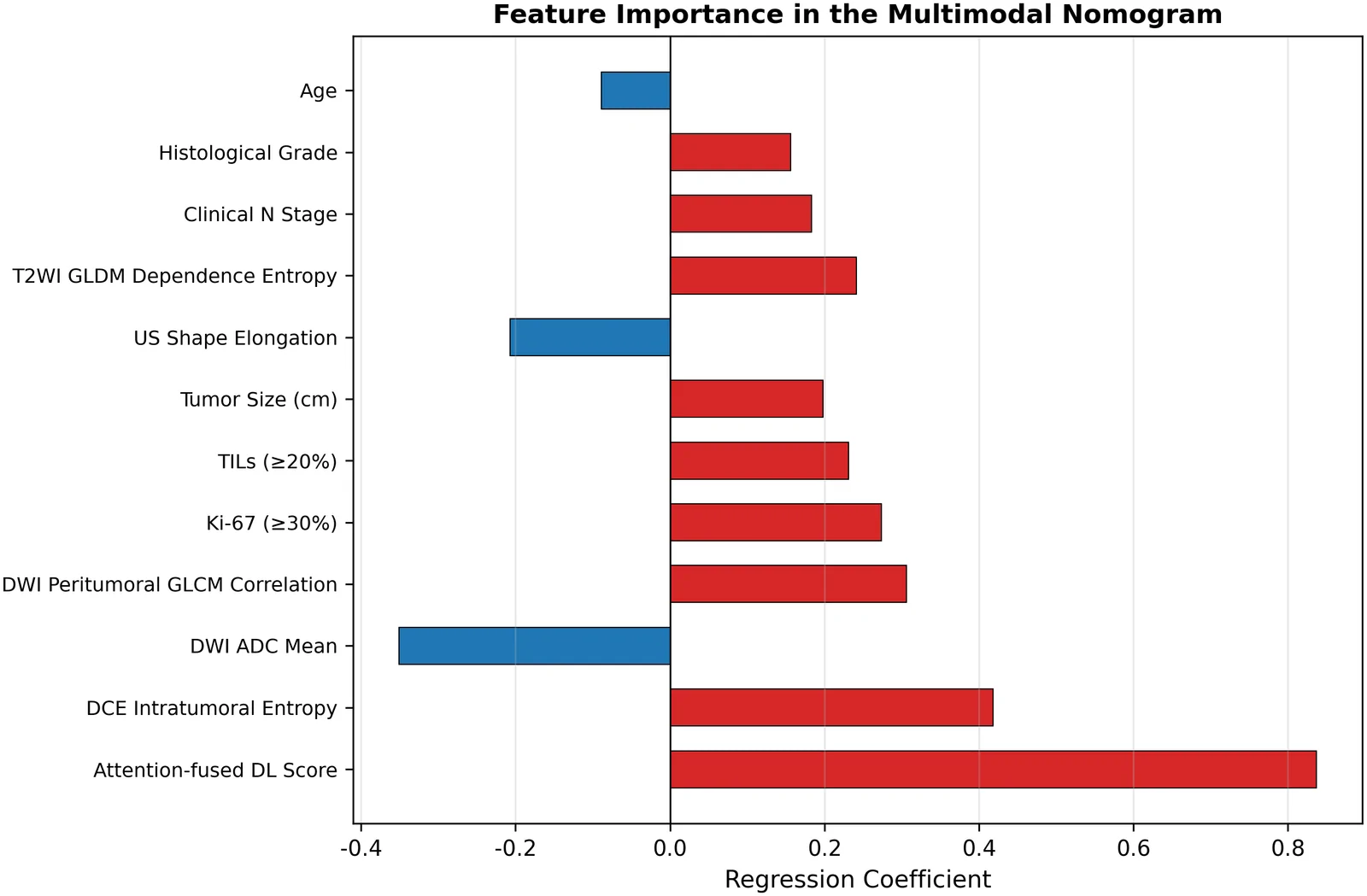
Feature importance (regression coefficients) of the variables incorporated into the multimodal nomogram.

### 3.5 Sensitivity and ablation analyses

At the Youden-index threshold, the nomogram correctly identified 26 of 34 pCR patients and 39 of 48 non-pCR patients (TP = 26, FP = 9, TN = 39, FN = 8). Detailed diagnostic performance at the locked threshold is reported in **Table 3**. Multi-threshold analysis showed that a high-specificity threshold of 0.65 yielded a positive predictive value of 0.85. Ablation analysis confirmed that the attention-fusion strategy outperformed simple concatenation (external AUC 0.856 vs. 0.837). Leave-one-center-out validation showed stable generalizability, with a mean AUC of 0.847 (range, 0.824-0.863).

**Table 3:** Diagnostic performance of the attention nomogram at the locked threshold in the external validation cohort.

Metric	Value (95% CI)
Sensitivity	0.765 (0.59-0.90)
Specificity	0.813 (0.67-0.92)
Accuracy	0.793 (0.69-0.88)
PPV	0.743 (0.57-0.87)
NPV	0.830 (0.69-0.93)
Positive likelihood ratio	4.08 (2.19-7.62)
Negative likelihood ratio	0.290 (0.14-0.53)
F1 score	0.754
Brier score	0.16
Calibration slope	0.93
Calibration intercept	−0.06

### 3.6 Hypothetical surgical planning analysis

In the external validation cohort, 34 patients achieved pCR. In a *post-hoc* analysis, the nomogram correctly classified 26 of these 34 patients as having a high probability of pCR, corresponding to 76.5% sensitivity among patients with confirmed pCR. Among these 26 patients, 13 had actually undergone mastectomy. Under a hypothetical model-assisted decision framework, these 13 patients might have been considered potential candidates for breast-conserving surgery, subject to standard anatomical, oncological, and patient-preference criteria.

We emphasize that this surgical-planning analysis is exploratory only. It is intended to illustrate the potential utility of preoperative pCR prediction as a source and should not be interpreted as evidence to directly support surgical de-escalation or changes in local treatment strategy.

In an exploratory analysis, 3-year disease-free survival did not differ significantly between patients who underwent breast-conserving surgery and those who underwent mastectomy (log-rank P = 0.77). However, this comparison was underpowered and confounded by selection bias inherent to the retrospective design. These findings should be interpreted with caution and cannot support changes in surgical practice without prospective validation.

### 3.7 Subgroup analysis

The nomogram maintained consistent performance across clinicopathological subgroups in the external validation cohort (**Figure 6**). AUCs ranged from 0.784 (histological grade G1-2, n = 21) to 0.893 (immunotherapy-containing NAT, n = 22). Performance was stable across Ki-67 strata (high: 0.867; low: 0.803), TILs levels (high: 0.884; low: 0.831), clinical T stages (T1-2: 0.861; T3-4: 0.798), and clinical N stages (N0: 0.879; N1-3: 0.826). The model showed relatively lower performance in the grade I-II subgroup (AUC 0.784, 95% CI: 0.612-0.931), likely reflecting the smaller sample size (n = 21) and the lower prevalence of high-grade features that drive model predictions.

**Figure 6:**
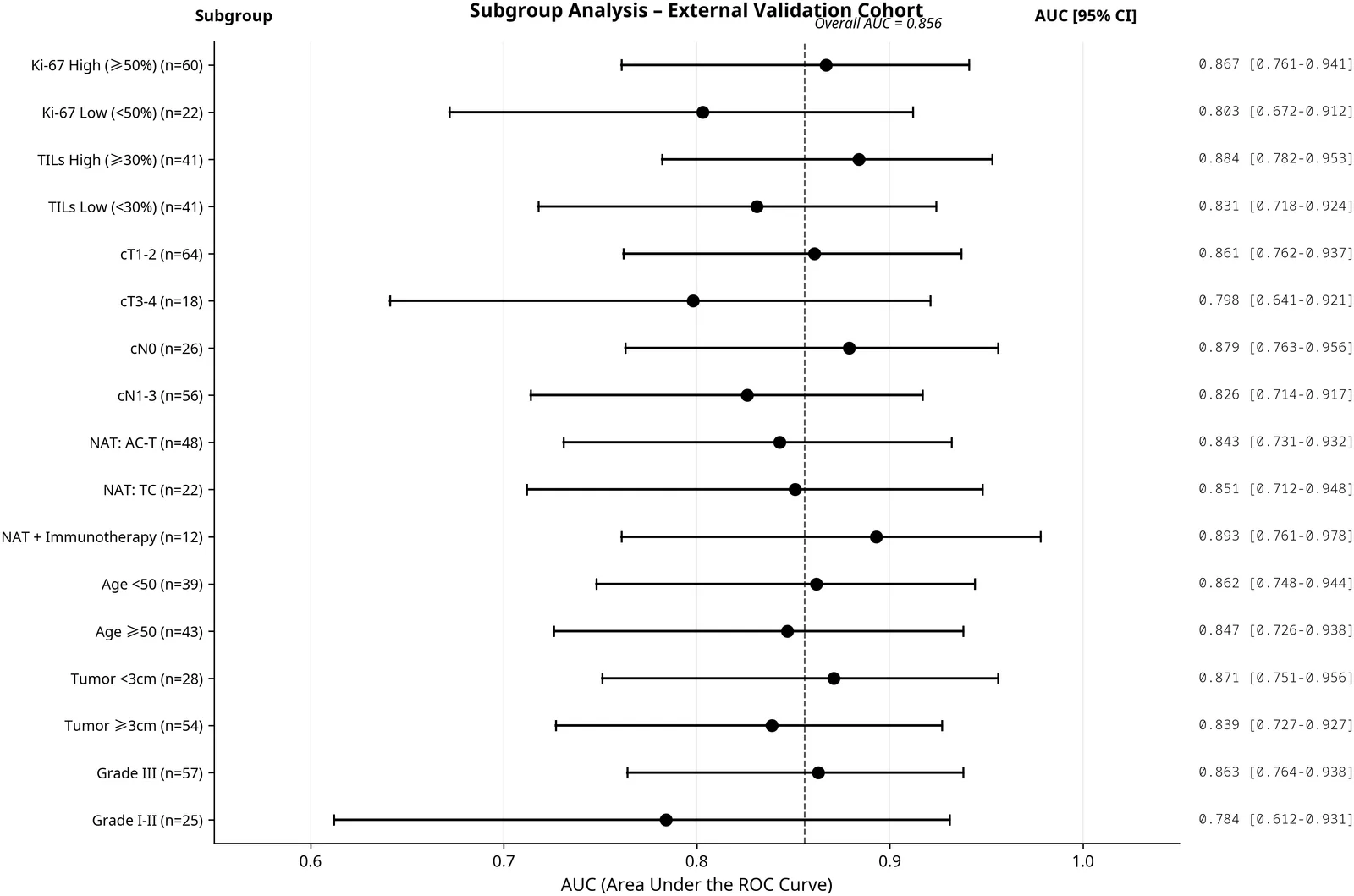
Forest plot showing the performance (AUC with 95% CI) of the multimodal nomogram across different clinicopathological subgroups in the external validation cohort.

## 4 Discussion

In this multicenter study, we developed and externally validated a multimodal model for predicting pathological complete response after neoadjuvant therapy in triple-negative breast cancer. The main finding is that integrating imaging-derived signatures with clinicopathological information provided better discrimination, calibration, and clinical utility than any single-modality approach, with preserved performance in an external cohort. This has clinical relevance because pCR is related to long-term prognosis and is increasingly used to guide treatment escalation or de-escalation after NAT. A reliable prediction tool may help identify patients who are less likely to respond to the planned regimen, support patient counseling, and improve multidisciplinary planning before surgery. Overall performance across all three cohorts and all evaluated model variants is compared in **Figure 7**, and the final integrated model is presented as a clinically interpretable nomogram in **Figure 8**.

**Figure 7:**
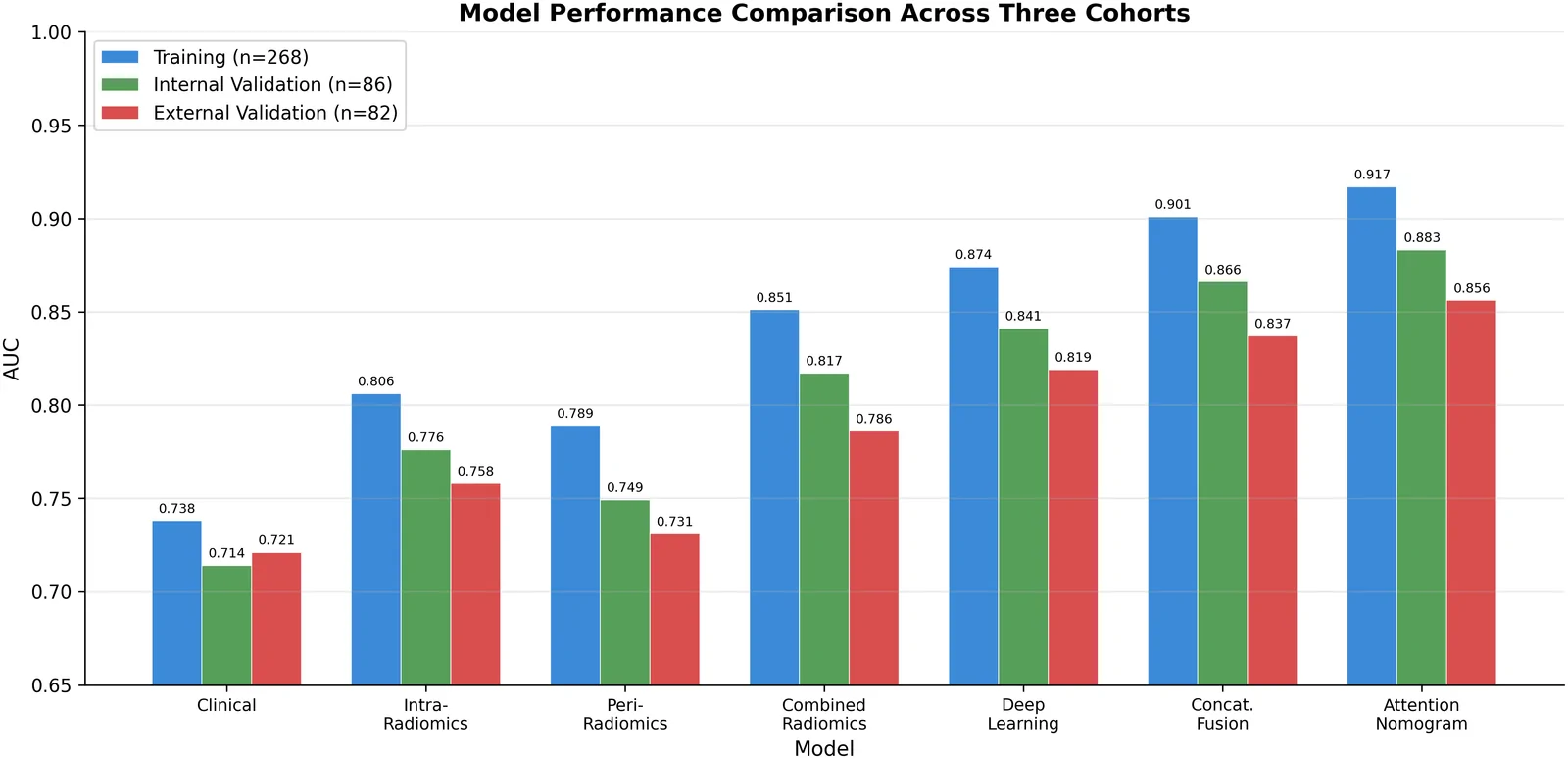
Comparison of AUC values across all cohorts for all evaluated models including ablation variants.

**Figure 8:**
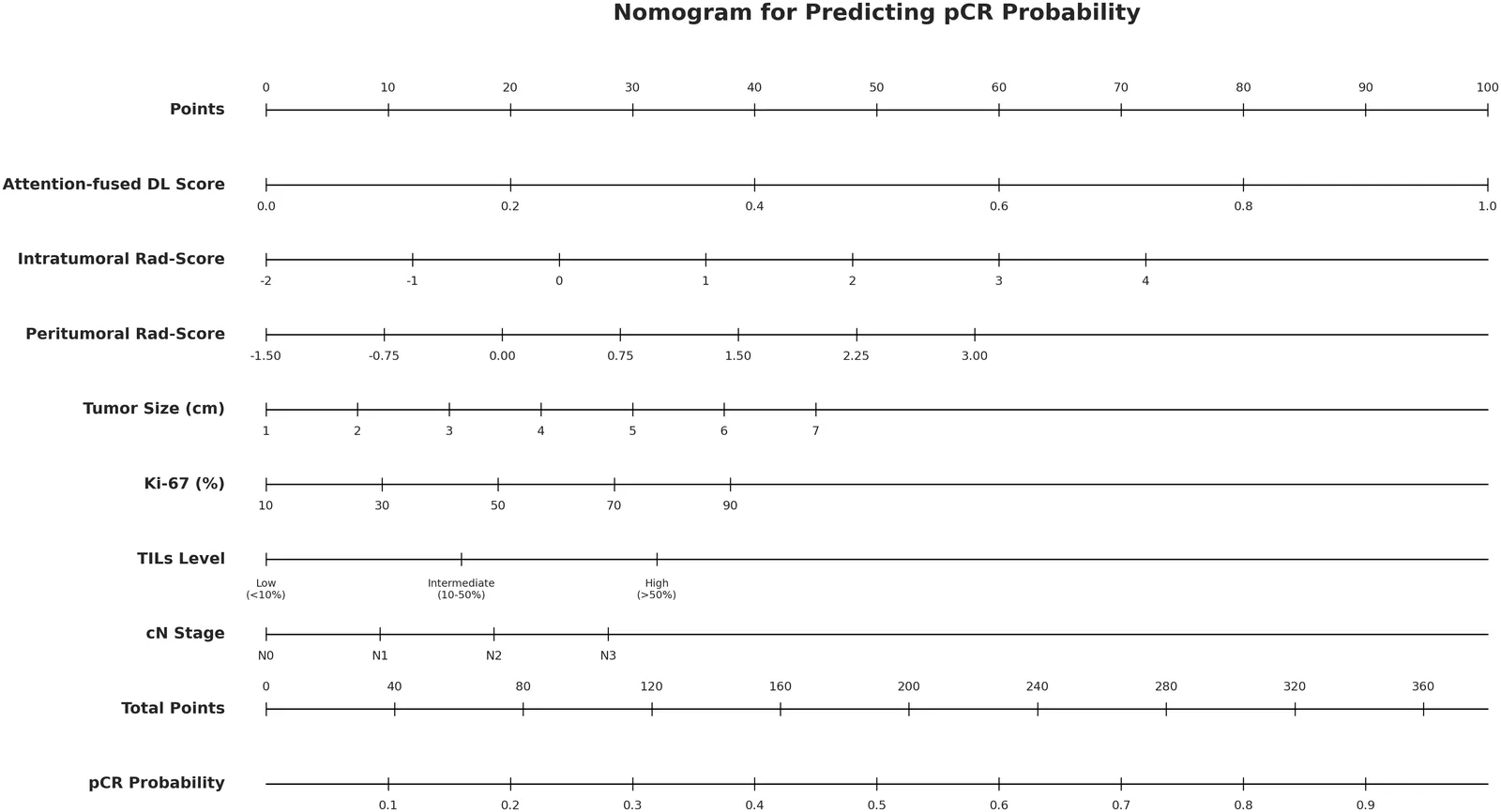
Nomogram for predicting pCR probability based on the integration of imaging and clinicopathological features.

Our findings are consistent with the growing literature showing that radiomics and deep learning can predict treatment response from pretreatment images. Jiang et al. reported a deep learning radiomic nomogram for locally advanced breast cancer with a validation AUC of 0.94 (8). Shi et al. showed that MRI-derived intratumoral heterogeneity achieved AUCs of 0.83-0.87 in external test sets (7), while the BMMR2 Challenge reported top models with AUCs of 0.803-0.840 (6). In triple-negative disease, Mohamed et al. found that DCE-MRI and DWI radiomics achieved a testing AUC of 0.802 (4), and Zhang et al. confirmed consistent predictive value in a meta-analysis (3). Ultrasound-based multimodal approaches have also performed well: Lyu et al. reported an external AUC of 0.84 in TNBC (1), whereas Wu et al. and Wang et al. reported AUCs near or above 0.89 (15, 16).

The superiority of our fused model parallels studies that explicitly evaluated multimodal learning. Mao et al. (10) developed MIFAPS, achieving AUCs of 0.932 internally, 0.882 externally, and 0.909 prospectively (10). Joo et al. reported that combining clinical data with MRI improved AUC from 0.827 to 0.888 (9). Liu et al. found that a comprehensive model incorporating mammography, ultrasound, MRI, whole-slide images, and clinicopathological features outperformed individual modalities (17). Guo et al. showed that cross-modal integration of pathology and temporal ultrasound achieved robust pCR prediction (12).

Recent studies have further highlighted the value of explainable AI in breast cancer modeling. Sha et al. (18) demonstrated that integrating deep learning features from mammography with SHapley Additive exPlanations (SHAP) values enabled reliable prediction of over 5-year recurrence of breast ductal carcinoma *in situ* post-lumpectomy. Yuan et al. (19) developed an XGBoost-based model incorporating systemic inflammation-nutrition biomarkers to predict neoadjuvant chemotherapy response in 3, 036 breast cancer patients, with SHAP analysis revealing ER status, HER2 status, and albumin as the most important predictive features. In addition, machine learning-based approaches have shown promise for prognostic modeling and novel subtype identification (20) as well as identification of epigenetically modified tumor cell subsets as prognostic and therapeutic targets (21). These studies collectively support our approach of combining multimodal imaging with explainable AI methods to enhance clinical interpretability. 

Recent studies further demonstrate the breadth of imaging-based breast cancer modeling, including radiomics of the tumor immune microenvironment and immunologic phenotypes (22, 23), and deep learning across breast MRI, PET/MRI, mammography, and histopathology (24–28). Radiomic and nomogram approaches have also been applied to recurrence, neoadjuvant response, and multimodal MRI or ultrasound analysis (29–36). Multiparametric radiomics has been used to characterize tumor heterogeneity, molecular subtype, nodal status, and prognosis, while recent reviews and explainable frameworks emphasize transparency and interpretability (37–46). Studies using early or posttreatment MRI, multimodal or federated learning, pretrained convolutional networks, histology, and nomograms similarly support treatment-response and outcome modeling, complemented by systematic work on postoperative outcomes and response biomarkers (47–58). Additional work on surgical margins, axillary response, molecular subtypes, and time-dependent multicenter validation suggests potential extensions of this framework (59–62).

From a biological perspective, there are reasons to explain why combining multiple imaging modalities may outperform a single data source. pCR is not determined by tumor size alone; it reflects the interaction between tumor cell-intrinsic chemosensitivity, drug delivery, vascular permeability, immune activation, stromal composition, hypoxia, and clonal heterogeneity. Contrast-enhanced MRI reflects vascularity and permeability, diffusion imaging is linked to cellularity, and ultrasound texture can reflect stromal architecture and acoustic heterogeneity. These data are complementary rather than duplicated. Fusion may strengthen common response-related signals and reduce noise from individual modalities, segmentation variability, and scanner effects. We extended previous efforts by incorporating both Grad-CAM++ heatmaps (**Figure 9**) and SHAP summary plots to demystify the “black box” nature of deep learning, revealing that the model’s attention was appropriately focused on the tumor core in DCE-MRI and the tumor periphery in DWI, aligning with known pathophysiological changes during NAT. In summary, the explainability analyses confirmed that the model relies on biologically plausible imaging features and clinically relevant variables, supporting its potential as a transparent decision-support tool.

**Figure 9:**
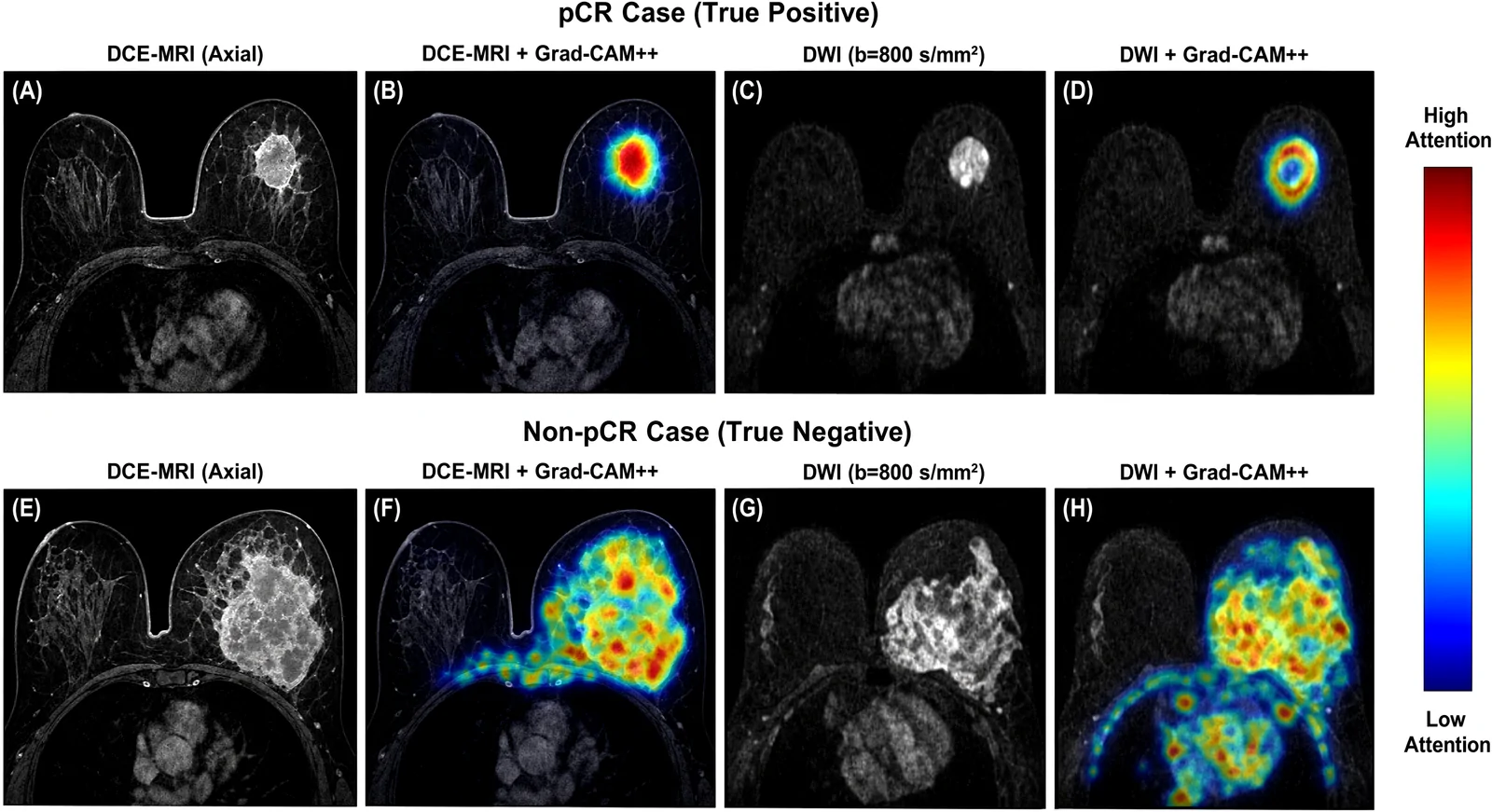
Grad-CAM++ attention heatmaps for representative cases. **(A-D)** pCR case (true positive): **(A)** axial DCE-MRI, **(B)** DCE-MRI with Grad-CAM++, **(C)** DWI (b = 800 s/mm²), and **(D)** DWI with Grad-CAM++. **(E-H)** non-pCR case (true negative): **(E)** axial DCE-MRI, **(F)** DCE-MRI with Grad-CAM++, **(G)** DWI (b = 800 s/mm²), and **(H)** DWI with Grad-CAM++. The color scale denotes model attention from low (blue) to high (red).

While the nomogram clearly outperforms the clinical model (external AUC difference of 0.135), the incremental gain over the radiomics-only model (AUC difference of 0.070) and the deep learning-only model (AUC difference of 0.037) is more modest, and the latter comparison did not reach statistical significance after Bonferroni correction. Therefore, claims regarding immediate clinical implementation should be viewed cautiously, and the added value of multimodal fusion over individual advanced imaging models requires confirmation in larger prospective cohorts.

If confirmed in prospective studies, this model could potentially be incorporated into the multidisciplinary workflow before definitive surgery. Patients predicted to have low probability of pCR might be considered for intensified regimens, early response imaging, or enrollment in adaptive trials. Patients predicted to be highly responsive could be prioritized for breast-conserving strategies or axillary de-escalation trials, provided that standard pathological confirmation remains mandatory. We emphasize that the model should serve as a risk-stratification aid rather than a replacement for clinician judgment or postoperative pathology. The exploratory surgical-planning analysis presented herein does not constitute evidence for changing current surgical practice and should not be overinterpreted as direct support for surgical decision-making.

This study has several strengths, including its multicenter design, independent external validation, standardized preprocessing, segmentation quality control, comprehensive model comparison, calibration assessment, and reporting structured according to TRIPOD principles.

Several limitations should be acknowledged. First, the retrospective design may introduce selection bias and unmeasured confounding. Second, the external cohort was relatively small (n = 82), which widens confidence intervals. Third, lesion segmentation was manual, making the workflow labor-intensive. Fourth, all patients were drawn from a Chinese population, and calibration may differ in other ethnic groups. Fifth, the surgical analysis was hypothetical and not prospectively validated. Sixth, NAT regimens were heterogeneous, and differences in chemotherapy backbone, platinum addition, immunotherapy combination, and dose intensity could affect pCR probabilities. Finally, residual variability from imaging equipment and acquisition parameters may have influenced performance despite harmonization efforts. We did not employ external datasets from different countries, and our sample size, though adequate for model development, may be insufficient to fully capture the extreme heterogeneity of TNBC. Future work should prioritize prospective, multi-institutional validation with integrated spatial transcriptomics to further elucidate the biological underpinnings of the radiomic features identified here.

Future research should shift the focus from prediction accuracy toward demonstrating clinical utility through prospective multicenter trials with locked models, predefined thresholds, and patient-important endpoints. Automated segmentation, integration with genomics and circulating tumor DNA, and federated learning approaches could further improve transportability and clinical applicability.

## 5 Conclusion

This multicenter study shows that a multimodal model integrating imaging-derived and clinicopathological information can predict pCR after NAT more accurately than single-modality approaches and maintains promising performance in external validation. Clinically, the model may eventually help identify patients who require treatment intensification or who may be candidates for carefully designed surgical de-escalation studies. At present, its role should be regarded strictly as an aid for pCR prediction and preoperative stratification rather than as direct support for clinical decision-making; prospective validation in larger and ethnically diverse cohorts is essential before clinical implementation.

## Data Availability

The original contributions presented in the study are included in the article/supplementary material. Further inquiries can be directed to the corresponding author.
